# Centralized and Federated Models for the Analysis of Clinical Data

**DOI:** 10.1146/annurev-biodatasci-122220-115746

**Published:** 2024-07-24

**Authors:** Ruowang Li, Joseph D. Romano, Yong Chen, Jason H. Moore

**Affiliations:** 1 Department of Computational Biomedicine, Cedars-Sinai Medical Center, Los Angeles, California, USA; 2 Institute for Biomedical Informatics, University of Pennsylvania, Philadelphia, Pennsylvania, USA; 3 Department of Biostatistics, Epidemiology, and Informatics, University of Pennsylvania, Philadelphia, Pennsylvania, USA

**Keywords:** centralized model, federated model, federated learning, electronic health record, EHR, clinical data, data analysis, data integration

## Abstract

The progress of precision medicine research hinges on the gathering and analysis of extensive and diverse clinical datasets. With the continued expansion of modalities, scales, and sources of clinical datasets, it becomes imperative to devise methods for aggregating information from these varied sources to achieve a comprehensive understanding of diseases. In this review, we describe two important approaches for the analysis of diverse clinical datasets, namely the centralized model and federated model. We compare and contrast the strengths and weaknesses inherent in each model and present recent progress in methodologies and their associated challenges. Finally, we present an outlook on the opportunities that both models hold for the future analysis of clinical data.

## INTRODUCTION

Central to working with biomedical big data is a database to store and manage biological and physical measures of organisms and humans. Databases come in all shapes and sizes and can be structured (e.g., MySQL and Oracle), semistructured (e.g., Neo4j and MongoDB), or unstructured (e.g., Amazon S3 and Google Cloud Storage). Each of these database options has advantages and disadvantages. For example, structured relational databases can be easily queried using tools such as Structured Query Language (SQL) but can be time-consuming to set up and modify for new data elements. Semistructured and unstructured databases are much more flexible but can be harder to query. Deciding on a database solution can be challenging in the biomedical domain because data can be very complex with many different modalities. Different modalities can be structured or unstructured, which can create challenges for data processing, data entry, data integration, database design, and, of course, constructing powerful queries.

Setting up and validating a database are just the beginning of a big data journey. A good database must be secure from those who should not have access to the data. It should also be user-friendly without unnecessary barriers. There can also be issues related to software costs, data storage costs, personnel costs, and data upload and download efficiency. Moving a database from the design stage to routine use can take years to complete, requiring significant financial resources.

Databases are the lifeblood of biomedical informatics and data science. One of the oldest examples of a publicly available database is GenBank, which was developed in the late 1970s and early 1980s for storing and making available nucleic acid sequences (1). GenBank stores each sequence in a single file with a specific accession number along with annotations about its location in the genome, the species it was derived from, and other information such as where it was first published. GenBank is a nice example of a semistructured database because each data file is independent but has a standard format for sequence and annotations, which can be easily queried. Relational or structured databases for molecular sequences have also been developed. Bio-Strings is a recent example (2).

One of the most ambitious biomedical database efforts has been the development and clinical application of electronic health records (EHRs) for storing and managing patient data for the purpose of informing healthcare decisions and billing for services (3). One of the earliest EHRs used the Massachusetts General Hospital Utility Multi-Programming System (MUMPS or M) programming language to access clinical data stored in multidimensional arrays (4). Interestingly, Epic, one of the largest commercial EHR vendors, uses MUMPS to access clinical data in its database. These efforts, and many others, along with policies such as meaningful use (5), have resulted in the widespread adoption of EHRs for patient care. Concurrent with these developments has been the rise of translational research enabled by access to clinical data stored in structured and semistructured EHR databases.

A key translational research area made possible by EHRs is precision medicine, which has the goal of identifying the right risk prediction models, diagnostic tests, procedures, and treatments for each patient based on their own unique medical history and constellation of biomarkers and environmental exposures. However, the EHR is not enough by itself. We need to measure biomarkers from the genome, metabolome, microbiome, etc. and integrate these data with clinical data to be able to identify key patient subgroups requiring unique clinical care.

**Figure 1:**
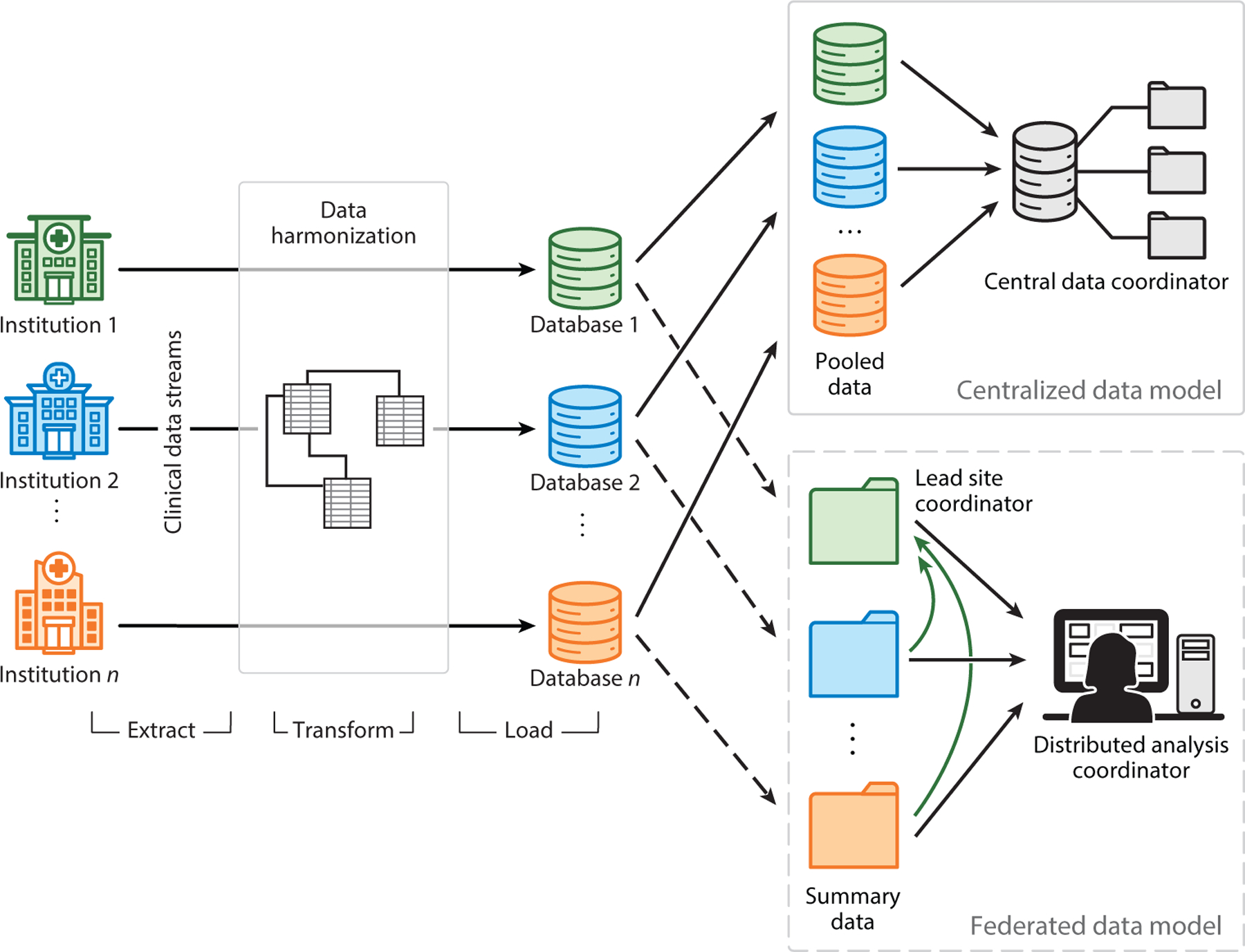
Overview of centralized and federated data models. Clinical data from multiple institutions first undergo extraction, transformation, and loading steps to prepare them for analysis. In the centralized data model, data from various institutions are pooled by the central data coordinator. In contrast, the federated data model only requires the transmission of a data summary, such as summary statistics and model parameters, for subsequent analysis. The analysis can be conducted either by a distributed analysis coordinator or by one or more participating sites.

Given the number of clinical, environmental, and omics biomarkers, precision medicine research requires large sample sizes, often larger than the available sample size. This is especially true for rare diseases. One solution is to aggregate data from multiple clinical sites in a centralized database, which is then made available to participating researchers. This is appealing because the data are all integrated in one place, providing a convenient source for queries. However, as the use of EHR data for research purposes expands, there are very real concerns about data security and patient privacy. Security and privacy issues include ransomware attacks, malware, denial of service attacks, phishing, breaches of confidentiality, and unauthorized access (6). An alternative approach is to keep the data locally at each of the participating institutions, thus requiring local analysis. This federated database approach provides more data security but requires special methods for integrating the analytical results across sites. Both approaches require the data to be mapped to a common data model (CDM) to ensure that all the data elements mean the same thing. The purpose of this review is to compare and contrast centralized and federated approaches to data management for precision medicine research (Figure 1).

## CENTRALIZED DATA MODELS

Centralized approaches to clinical data sharing—and research artifacts that are derived from those clinical data—are defined by aggregating and maintaining the entirety of the included data in a single location. Location in this context can mean either a server address or a geographic location, and from an implementation perspective the distinction has major implications for data security, but for the sake of this review we do not need to differentiate between the two. The approach can involve databases from either a single site (usually a hospital) or multiple sites (several hospitals with a shared set of research collaborators or a group of healthcare sites that are members of a larger healthcare network). To look at the current state of centralized approaches to EHR research for precision medicine, we consider specific examples of two separate artifacts of clinical informatics research: (*a*) CDMs and (*b*) ongoing initiatives that provide centralized datasets for networks of researchers.

### Common Data Models

Raw observational data collected from any clinical environment will be structured in a way that reflects the processes, policies, and procedures of the institution. These are sources of bias (7, 8) that should be considered and/or corrected to uncover the true patterns of human health that are present in the data. For example, site-specific healthcare processes and cultural biases are a leading contributor to missingness within EHRs, thus significantly impacting their ability to yield realistic predictive models and generalize to disadvantaged groups (9). A popular and effective method to control for this bias is to externally define a collection of standardized data schemas that are designed for biomedical research rather than health system processes (10). These standardized sets of schemas are called CDMs, and they represent one of the leading ways that electronic health and biobank data are converted for secondary use while mitigating certain sources of bias (11); they are also used in fields beyond biomedicine. There are numerous diverse CDMs for EHR data that have been developed in recent years, but only a small number represent the vast majority of CDM use worldwide (12). Here, we focus on three of the most popular to highlight major design principles: the Observational Medical Outcomes Partnership (OMOP) CDM, the Sentinel CDM, and the Patient-Centered Outcomes Research Network (PCORnet) CDM.

One of the earliest and most successful major CDM is the OMOP CDM, which was defined by the Observational Medical Outcomes Partnership, a former public/private partnership that included members from the US Food and Drug Administration (FDA), pharmaceutical companies, and healthcare providers (10). In recent years, OMOP has been succeeded by the Observational Health Data Sciences and Informatics (OHDSI) collaborative that continues to develop the CDM (currently standardized at version 5.4) under the original OMOP name (13). One of OMOP/OHDSI’s central goals has been the harmonization of disparately structured data into a single, contiguous, meaningful format, and the group has accordingly applied the CDM to numerous national and international clinical datasets (11, 14). The OMOP CDM defines a series of schemas for relational databases that are divided into six categories: clinical data, health system data, standardized vocabularies, health economics data, derived elements, and metadata. Three tables in particular act as hubs linking most of the other tables; these are PERSON, VISIT_OCCURRENCE, and CONCEPT. The PERSON table acts as the central identity management for all persons in the database, storing a unique person_id and various demographic features describing each person. VISIT_OCCURRENCE links a person to a series of events (defined in tables such as DRUG_EXPOSURE, MEASUREMENT, PROCEDURE_OCCURRENCE, and others) that occur over the duration of an interaction with the healthcare system, and each visit is identified by a unique visit_occurrence_id. CONCEPT defines fundamental units of meaning used to express clinical information in all domain tables and includes diverse entities like drugs, diseases, procedures, demographic concepts, and many others. Most clinical data tables include columns for patient_id, visit_occurrence_id, and at least one concept_id. Drug exposures are separated into single exposure events (DRUG_EXPOSURE) and extended periods of exposure (DRUG_ERA), which are intended to facilitate time series analyses (15). The OMOP CDM was not explicitly designed to handle multiomics or biospecimen data, but several proof-of-concept studies have shown that it is possible to include these data in the CDM (16). Generally, these data are collectively maintained in biobanks, which are large-scale biorepositories managed by either a healthcare institution (such as a hospital or hospital system) or a larger, often governmentally funded, public health initiative (such as the All of Us or UK Biobank program). The Penn G&P (Penn Genotype & Phenotype) data repository is one example of the effective integration of biobank data into an EHR-linked clinical database based on the OMOP CDM (17). Under OHDSI, the OMOP CDM is shifting toward increased use under a federated data paradigm, as discussed below.

Another major example of a clinical CDM is the one defined by the Sentinel Initiative, which is an effort coordinated by the FDA to improve the safety of medicinal drugs via postmarketing surveillance (18, 19). The Sentinel CDM acts as a data template, where the initiative centrally defines programs to run on clinical data with the CDM in mind, and Sentinel Data Partners then run the programs on a copy of their data (potentially from multiple sites) populated into the CDM. Compared to the OMOP CDM, the Sentinel CDM places more emphasis on ancillary and administrative claims data and focuses heavily on drug exposures and diagnoses. It also defines tables for self-reported patient data (20), which are absent from the OMOP CDM definition. Like OHDSI/OMOP, the Sentinel project increasingly encourages the use of federated tools to aggregate data from multiple sites—briefly, each partner retains their dataset locally, while the FDA distributes a query to be run on the local site-specific instances of the CDM, the results of which are then securely returned to the FDA, and the results from multiple sites are combined. The Sentinel Initiative has also led to a spinoff named FDA-Catalyst that augments the Sentinel data with patient/provider interaction data, meant to enhance clinical trial study design (21).

A third popular CDM is the one defined by PCORnet, a national group of eight clinical research networks (CRNs), each of which includes at least two major healthcare systems (22). The PCORnet CDM allows each CRN to aggregate the data from its network of healthcare sites into a single database for the purpose of large-scale EHR studies, the results of which can be compared across the CRNs. In its current revision (version 6.0), the CDM is a single set of tables that collects various types of clinical concepts and annotates them with IDs identifying unique patients (PATIDs); these tables include ENCOUNTER, DIAGNOSIS, PROCEDURES, CONDITION, DEATH_CAUSE, PROVIDER, and others. The domain focus of the CDM is like that of OMOP, but with a comparatively simpler structure. A notable omission is a lack of tables for aggregating conceptual entities (like the OMOP CONCEPT and VOCABULARY tables), where vocabulary standards are encoded directly within the clinical data tables themselves. For example, in the DIAGNOSIS table, diagnosis codes are generally provided as either ICD (International Classification of Disease) or SNOMED (Systematized Medical Nomenclature for Medicine) codes, but PCORnet acknowledges that they are adding diagnoses defined in other vocabularies as those vocabularies become more widely used. The CDM does not have the capability to store data from biobanks, but it does provide an optional Boolean field to indicate whether biobank samples are available for a given patient.

It is important to note that all three of these examples of CDMs are coordinated by groups that originally designed them for centralized analysis of clinical data but are now shifting toward increased support for federated methods of analysis (23). For example, while the Sentinel CDM allows each data partner to run programs defined by the initiative on their centrally aggregated data, the initiative also manages a distributed metadatabase infrastructure named the Sentinel Distributed Database, where the Sentinel Operations Center (SOC) distributes queries to each data partner, the data partners run those queries on their own centralized implementations of the CDM, and the results are then transmitted securely back to the SOC for aggregation and analysis (24). There are still many ongoing studies that stick exclusively to the centralized approach for analyzing CDM-compliant data, though it may be impossible to detect associations [e.g., genome-wide association studies (GWASs) on rare events or with alleles that have small effect sizes] unless a federated approach is used, due to sample size limitations.

### Major Research Efforts Using the Centralized Data Approach

Beyond CDMs, there are distinct research initiatives that aggregate large quantities of observational health data and make the resulting dataset available for secondary research use. These can either reuse data collected from multiple health systems (e.g., academic medical centers) or use data collected directly by the research initiative itself. They can be for general use (i.e., not targeting a specific disease or demographic) or focused on a particular health initiative. As they follow a centralized rather than a federated paradigm, these research efforts make their entire data collection available to users who gain appropriate access, generally by being members of credentialed academic or other research institutions and by passing an application process. For the sake of patient privacy, some of these efforts obscure access to the raw data and instead provide a restricted interface where users either make queries or submit higher-level requests, which are then executed on secured remote servers and the result is returned to the researcher (25). To illustrate the variety of these research efforts, we look at three popular examples in greater detail.

The Electronic Medical Records and Genomics (eMERGE) network is one of the first major initiatives to provide EHR data linked to well-curated, large-scale biobank data at a national scale (26). eMERGE is funded and coordinated by the US National Institutes of Health (NIH)/National Human Genome Research Institute and includes data from 11 major participating sites. Researchers at the participating sites also collect data from other medical centers and incorporate them into the network. eMERGE is also a leading developer of electronic phenotypes—algorithms for selecting patients in an EHR database with a certain condition. eMERGE-developed EHR phenotyping algorithms are deposited in the Phenotype KnowledgeBase (PheKB) (27) and are generally accessible to the public. The eMERGE dataset has resulted in 771 publications at the time of writing, many of which are large-scale GWASs or, more recently, studies to estimate polygenic risk scores.

While eMERGE is a governmental and academic partnership, the Cosmos dataset is assembled by Epic, a commercial entity that is the largest EHR vendor in the United States. Epic promotes Cosmos as “the largest integrated database of clinical information in the United States,” with 162 million patient records spread over 5.7 billion clinical encounters, sourced from 22,500 health clinics and 1,063 hospitals (28). As of February 2022, more than half of Epic’s customer base has signed up to contribute data to Cosmos. To protect patient data, all records are fully deidentified at the level of the source clinics before being aggregated into the dataset. A long-term goal of Cosmos is to produce tools that allow clinicians to ask questions of the dataset in real time during patient encounters to assist in treatment decisions. Researchers who sign up for Cosmos access can run analytical queries via a centrally hosted instance of Epic’s SlicerDicer tool. As a corporate product, Epic adds value to Cosmos by regularly rolling out new tools to access and query the data, such as clinical trial matchmaking functionality. Research on the dataset is coordinated by the Epic Research public benefit corporation, and results of studies are released publicly on the website (https://www.epicresearch.org).

The National COVID Cohort Collaborative (N3C) is an effort from NIH’s National Center for Advancing Translational Sciences to assemble a large cohort specifically intended for COVID-19 research. The number of patients in the datasets is continually increasing and at the time of writing includes 16 million unique patients from 76 clinical sites, 6.6 million COVID-19 cases, and 19.2 billion rows of clinical data (29). Data are available in decreasing levels of anonymization to user types with increasing levels of institutional credentials. N3C tracks the various research projects (currently 401) that are either in progress or completed, and it maintains a catalog of peer-reviewed publications (currently 101) directly resulting from access to the datasets. Data are provided in both JSON ( JavaScript Object Notation) and CSV (comma-separated values) formats and are accompanied by a browser-based dashboard showing public summary statistics and other information about the datasets.

### Research Methods Used with the Centralized Data Approach

Methods appropriate for performing research on centralized clinical data resources largely depend on the level of access that researchers have to the underlying data. When researchers can fully access the raw data elements (e.g., in flat-file or relational database formats), the methods are by and large the same as for any other type of statistical or epidemiological research. Since the efforts discussed above have generated incredibly large cohorts of patients that often include many geographical and socioeconomic contexts (particularly in datasets aggregated from many clinical sites), there are often extra statistical and interpretation considerations that need to be used to effectively learn real patterns in the data. These include heuristic and/or statistical imputation of missing data that are not collected consistently across sites (30), hierarchical modeling in which specific Bayesian parameters are learned for each population within a single analysis (31), and intentional reduction of interpopulation variance prior to analysis (32), among others. This is further complicated by the fact that large clinical data resources can report tens of thousands of features that can be incorporated into predictive models. For example, OHDSI’s Athena standardized vocabulary tool currently reports over 8.3 million distinct clinical concepts that can be present in an OMOP CDM–formatted database. As such, it is often necessary to explicitly apply feature selection or feature extraction techniques to identify a smaller set of variables in statistical or machine learning analyses on the database.

Patient heterogeneity and the high prevalence of covariates/confounders also mean that researchers should assess whether it is appropriate to construct balanced subcohorts prior to analyzing the data. The most common—and best mathematically supported—of these techniques is arguably high-dimensional propensity score matching, where a logistic regression model is trained to predict the probability of each patient being in the true case or control cohort based on all available predictive features, with the resulting probability scores applied by that trained model (called propensity scores) then used to match case patients to a proportional number of control patients (33). The resulting distributions of confounders can then be used to verify that the matching procedure was successful in eliminating disproportionate distributions of those confounding variables among cases and controls, thus mitigating the risk that any predictive model trained on the new subcohorts will erroneously train a model based on the presence/absence of the confounder(s). High-dimensional propensity score matching refers to the case when there are many predictive features being adjusted for in the matching process. When researchers have only limited access to the data—for example, through an analytics dashboard (e.g., TriNetX) or another remote querying interface (e.g., OHDSI’s ATLAS tool), rather than direct access to the underlying data (34, 35)—it is crucial to understand the capabilities of the interface and to rigorously define cohorts using the most sophisticated techniques available. These interfaces often allow users to define cohort inclusion and exclusion criteria in a way similar to the definitions of electronic phenotyping algorithms, so prudent use of predefined algorithms from PheKB or similar resources can be tremendously helpful in building high-quality patient cohorts through an analytics interface.

Not unique to the centralized approach—but still critical to discuss—is the need to always assess fairness while designing precision medicine studies (36). By fairness, we are specifically referring to the meaning of the term in relation to social justice. Traditionally, studies of human health have been overwhelmingly biased to benefit socioeconomically privileged groups, with the greatest benefit often being heavily skewed toward white male patients of European descent. Biomedical and health researchers have a moral and ethical mandate to eliminate this bias by intelligently designing studies in a way that equitably reduces health disparities. Fortunately, this has become a major topic of discussion in recent years, with prominent journals and academic institutions explicitly acknowledging this need, and ethical artificial intelligence (AI) becoming established as a high-profile field with rapid growth and funding opportunities. Nonetheless, there remains much work to be done.

## FEDERATED DATA MODELS

Over the past two decades, the number of research networks embedded in healthcare systems has grown significantly, including OHDSI, PCORnet, Sentinel Initiative, and the NIH-funded Health Care Systems Research Collaboratory, presenting ample opportunities for multicenter research. The onset of the COVID-19 pandemic further prompted the establishment of various national and international multi-institutional research consortia, such as 4CE (Consortium for Clinical Characterization of COVID-19) (37), the RECOVER initiative (Researching COVID to Enhance Recovery; https://recovercovid.org), and N3C (29), among others. However, much of the multicenter healthcare data is stored at different locations. Privacy regulations often safeguard patient-level information, rendering the sharing of such data across partners impractical or requiring substantial operational efforts. Federated learning models played a critical role in enabling collaborative modeling in multisite settings. We review different data architectures under which federated learning models are implemented. Subsequently, we delve into key considerations essential for effectively deploying federated learning models in real-world settings. Finally, we cover pioneering work in the last two decades and provide insights into ongoing efforts.

### Data-Sharing Architectures and Workflows of Implementing Federated Learning Algorithms in Distributed Research Networks

**Figure 2:**
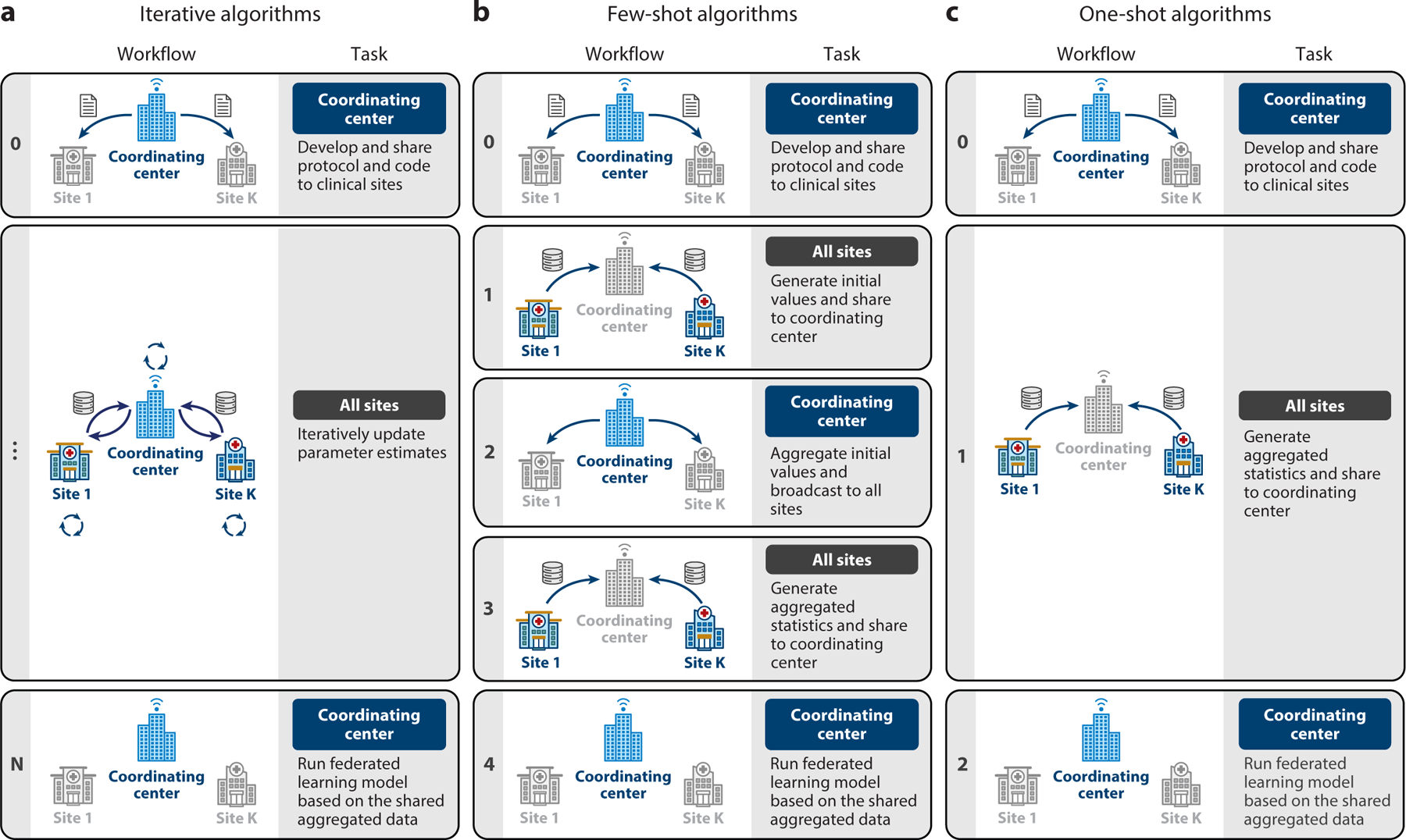
Three types of data-sharing architectures and workflows for running federated learning algorithms in real-world settings. (*a*) A coordinating center (or a lead site) facilitates iterative updates of the estimated model parameters via an automated web server that is connected with datasets from data partners. (*b*) A coordinating center (or a lead site) manually handles few-shot rounds of communications of aggregated data across data partners. (*c*) A coordinating center (or a lead site) shares a protocol with data partners for a one-shot contribution of their aggregated data.

Figure 2 illustrates three commonly encountered data-sharing architectures. In a federated learning model, there are typically two types of investigators. The first type belongs to the investigative team in the coordinating center, which is responsible for developing the analysis protocol. In some scenarios, investigators in one (or several) of the data sites take the lead in developing the analysis protocol, where they effectively act as the coordinating center to facilitate the federated analyses in a distributed research network. This includes defining scientific questions, study cohorts with clear inclusion/exclusion criteria, outcome variables, predictors or comparative groups, and type of models/algorithms used in the analysis. The investigative team is responsible for sharing the protocol, developing and testing the program of the federated learning algorithm, and coordinating communications among data partners. The second type of investigator comprises analytic teams at different data partners. Their responsibilities include preparing data (typically in a CDM), running the shared program of the federated learning algorithm, obtaining and reviewing results, and sharing results with a secure server provided by the coordinating center.

Figure 2a illustrates how a coordinating center facilitates iterative communications across data partners, allowing the updating of estimated model parameters until the algorithm converges. The number of iterations can vary based on data distribution and model complexity, ranging from a few dozen to hundreds of thousands. For this reason, such an iterative federated learning model is suitable for situations where a secure computing infrastructure is established with automated exchange of aggregated data across all data partners. Examples of algorithms in this setting include the Grid Binary Logistic Regression (GLORE) (38) and WebDISCO (39), a web service for distributed Cox model learning without patient-level data sharing.

An advantage of such a setting is that once such infrastructure is built, a large number of statistical and machine learning models can be implemented by iterative divide-and-conquer updates [e.g., Fisher scoring (40) or Newton–Raphson updates (41, 42)], and the results are often lossless (i.e., they obtain results identical to those from analyzing the pooled individual-level data). On the other hand, implementing such infrastructure across data partners can be logistically, administratively, and legally challenging. In addition to the need for an advanced secure computing environment, more importantly, the approach requires organizational trust and multi-institutional data usage agreements, which could take a long time to establish. For this reason, the scalability of this framework toward a large number of data partners is limited.

Figure 2b illustrates a few-shot federated learning setting where a coordinating center (typically) manually handles updates of model parameters by communicating across data partners. This framework avoids the need for demanding infrastructure for automated data exchange of aggregated data across all data partners and hence is applicable to broader collaborative modeling settings, such as international collaborations within the OHDSI. Protocols for federated learning in this setting are designed to be communication efficient, typically within two or three iterations. Examples of algorithms of this type include the one-shot distributed algorithm to perform logistic regressions (ODAL) (43, 44) and the one-shot distributed algorithm to fit a multicenter Cox proportional hazards model (ODAC) (45). Although ODAL can be implemented with a minimum of one round of communication, it is often advised that a meta-analysis should be implemented for more accurate initial values than the local estimates in order to get superior performance. Notably, the ODAC algorithm needs an initial step of sharing unique event times to construct a joint risk set at each event time over all subjects across data partners.

Algorithms with more than three iterations, such as the distributed penalized quasi-likelihood (dPQL) algorithm (46), could run into synchronization problems when updates of the estimated parameters need to wait until all data partners submit their results in the previous rounds. If one or more sites delay their updates at a given round of computing, the update toward the next round will be delayed and sometimes a decision at the coordinating center needs to be made whether to drop the delayed data partners. Given the synchronization problems, scaling this framework toward a large number of data partners is a challenge.

Figure 2c outlines the setting of one-shot federated learning, akin to the simplicity of traditional meta-analysis. In this setting, all data partners need to upload their aggregated data only once. Examples of one-shot algorithms include the distributed linear mixed model (DLMM) algorithm (47) and the Sum-Share algorithm for testing of pleiotropic effects using summary statistics (48). Unlike few-shot or iterative algorithms, one-shot algorithms do not require initiation of model estimation, which poses higher requirements on the algorithm design. On the other hand, one-shot algorithms are extremely applicable and scalable compared to the previous two frameworks. Specifically, since each data partner needs to run the algorithm only once and update the results, it has the same level of adoption as the commonly used meta-analysis. One-shot algorithms are expected to have the best scalability because they do not need infrastructure for the automated exchange of aggregated data, nor do they suffer from synchronization problems. Typically, a deadline for sharing aggregated data can be prespecified in the protocol, and only results uploaded before the deadline will be included in the federated learning model.

### Evaluation Metrics for Federated Learning Algorithms in Real-World Settings

The implementation of federated learning algorithms in real-world data settings involves multifaceted considerations encompassing privacy, communication constraints, statistical accuracy, sources of heterogeneity, and implementation readiness.

Each federated learning algorithm possesses specific requirements and properties, making it crucial to understand them when selecting the appropriate algorithm. Key factors to consider include privacy protection, statistical accuracy, communication efficiency, and heterogeneity awareness.

#### Privacy protection.

Federated learning models excel at avoiding the sharing of patient-level data. Different federated learning algorithms offer varying levels of privacy protection based on the collaboration network’s protocol. For example, PCORnet consortia like PEDSnet truncate cell counts fewer than 11 as “*n <* 11,” while some of the OHDSI data partners may share counts up to 6 and truncate as “*n <* 6.” Other consortia may demand higher levels of privacy protection, such as differential privacy (49, 50), homomorphic encryption (51), and multiparty encryption (52), among others (53). It should be noted that differential privacy methods may impact the model’s performance due to the introduction of noise. The extent of performance loss depends on the data-generating mechanism and the model type. In real-world settings, privacy considerations should be contextualized with regard to data-sharing, user access to shared (aggregate) data, and data-sharing agreements.

#### Statistical accuracy.

Federated learning algorithms often yield highly accurate results compared to the gold standard (i.e., results from analyzing the centralized data). It is important to quantify the difference between the results from a federated learning algorithm and the gold standard.

#### Communication efficiency.

The number of iterations in a federated learning algorithm significantly influences feasibility, required infrastructure, time expense, and scalability (in terms of the number of included data partners) in a federated learning study.

#### Heterogeneity awareness.

**Figure 3:**
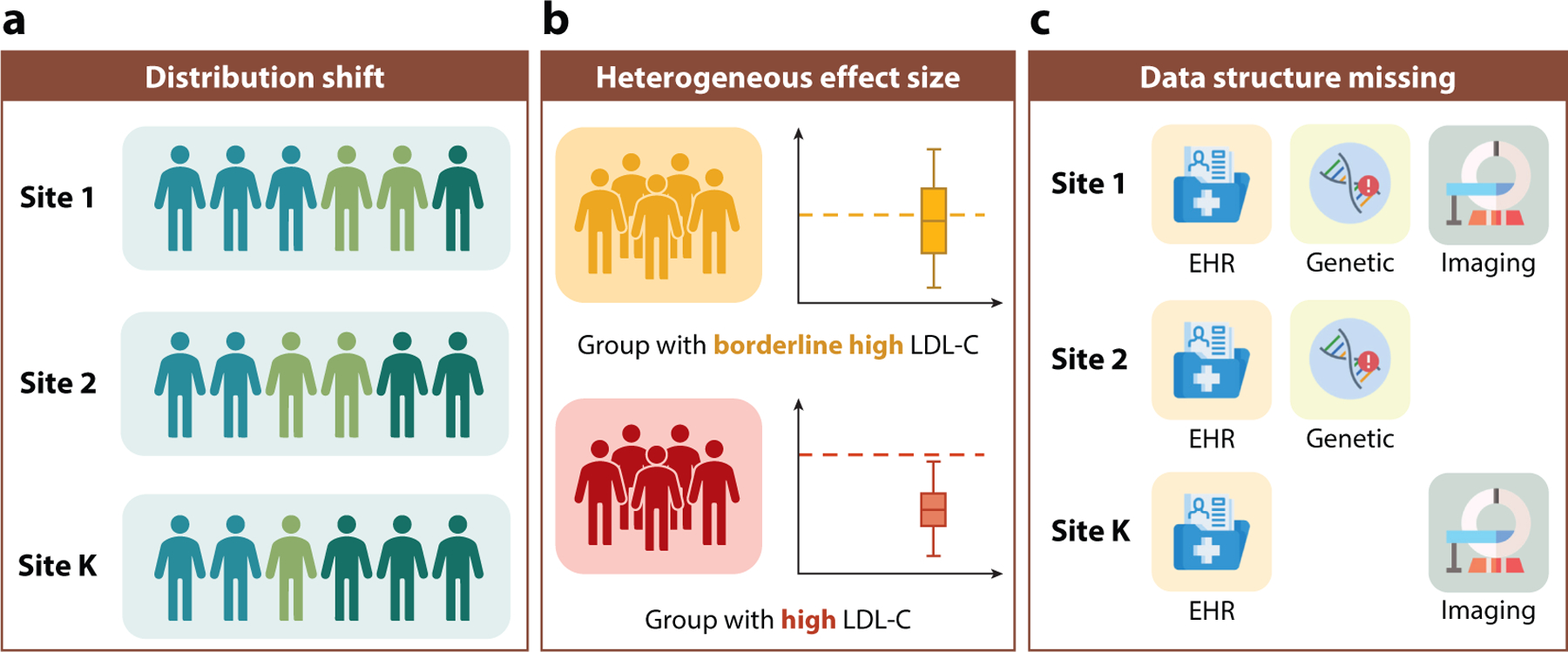
Illustration of three types of between-site heterogeneity. (*a*) A distribution shift due to intrinsic differences between subjects at different data sites. (*b*) A heterogeneity effect size due to effect modifications across sites. (*c*) A heterogeneous data structure due to a varying modality of data across sites. Abbreviations: EHR, electronic health record; LDL-C, low-density lipoprotein cholesterol.

Figure 3 illustrates various types of heterogeneity that could arise across decentralized data sites. The first type is a distribution shift, caused by intrinsic differences in characteristics of subjects in each data site, such as the race composition, age distribution, and differences in comorbidities. The second type is a variation in associations or effects across sites. Navarese et al. (54) conducted a meta-regression analysis of 34 randomized clinical trials, investigating the magnitude of reduction in mortality associated with low-density lipoprotein cholesterol (LDL-C) lowering. They found that more intensive LDL-C lowering therapy was statistically significantly associated with a progressive reduction in total mortality with higher baseline LDL-C levels; however, such a relationship was not present with baseline LDL-C levels less than 100 mg/dL. The third type of heterogeneity is in the data structure. Different data sites could have a subset of multimodalities of the features. For example, the UK Biobank contains a wide spectrum of features of patients in their database including EHRs and genetic and imaging data, whereas some other sites may have a subset of these modalities.

### pSCANNER as a Pioneering Work: Iterative Federated Learning Algorithms

In 2010, Lucila Ohno-Machado and her team at the University of California, San Diego, pioneered secure data sharing in real-world settings with the development of the Patient-Centered Scalable National Network for Effectiveness Research (pSCANNER) (55). This federated distributed research network, funded by the Patient-Centered Outcomes Research Institute, connected multiple clinical data partners in California. Leveraging ideas from parallel computing, pSCANNER enabled the connection of data from 21 million patients, allowing investigators to conduct multivariate statistical analyses by running privacy-preserving distributed computation models. Among the signature algorithms within pSCANNER are GLORE and WebDISCO, which are iterative federated learning algorithms for fitting logistic regressions and Cox proportional hazard models—two of the most widely used models in observational studies in biomedical sciences and public health.

While pSCANNER achieved great success in advancing our knowledge of several health conditions through the use of massive real-world data, including congestive heart failure, Kawasaki disease, and obesity, its reliance on iterative algorithms posed limitations. Scaling up proved challenging due to the substantial infrastructure required from participating data partners, the need for multi-institutional data usage agreements, and the establishment of organizational trust. As a result, this model faced constraints in application to distributed research networks lacking computational infrastructure for iterative communications, where aggregated data are often communicated via Secure Shell File Transfer Protocol (SFTP) or secure email communications.

### Recent Federated Learning Models in Distributed Research Networks

Recent efforts in federated learning models have predominantly focused on few-shot and one-shot algorithms due to their broad applicability. Notably, the seminal work on surrogate likelihood framework (56–58) for communication-efficient distributed inference has motivated a sequence of few-shot federated learning algorithms for various types of outcomes, including binary outcomes (43, 44), count outcomes with overdispersions (59), zero-inflated outcomes (60), and time-to-event outcomes (45). These algorithms, applicable with minimal rounds of communication across data partners [i.e., (*a*) use the local estimate (i.e., fitting the model at a data site) as the initial value, (*b*) broadcast a program with the initial value to all other sites, and (*c*) construct the surrogate likelihood at the local site to obtain the final estimate] have shown superior performance compared to meta-analysis methods, particularly in scenarios with rare outcomes or unbalanced covariate groups. These advantages make these algorithms particularly suitable for pharmacoepidemiologic and pharmacovigilance studies when rare adverse events are of primary interest. However, the sensitivity of results to the choice of the local site generating initial values poses a challenge. To enhance statistical accuracy, it has been proposed to use a meta-analysis estimate as the initial value, requiring three rounds of communication for initialization, estimation at each site, and result aggregation (45). In these cases, three rounds of communications across data partners are needed, including initialization (i.e., obtaining meta-analysis estimates as the initial values), estimation at each site (i.e., obtaining surrogate likelihood estimates at each site), and aggregation (i.e., weighted averaging the surrogate likelihood estimates across sites).

An important challenge in both centralized data models and federated data models is to properly account for between-site heterogeneity. As illustrated in Figure 3, various types of heterogeneity should be considered. Notably, a new surrogate efficient score-based framework has been proposed to integrate data from heterogeneous distributions where the effect sizes can be different across sites (61). In parallel, built on top of conditional inference and U-statistics (62), federated learning algorithms that handle site-specific incidental parameters have been developed (63). For time-to-event outcomes, federated learning algorithms have been developed that allow for site-specific baseline hazard functions, which offer great flexibility in modeling heterogeneous populations (64, 65). Thanks to the additivity of the partial likelihood for the stratified Cox model, this heterogeneous algorithm avoids the need to construct the shared risk set, which is in fact more communication efficient than the corresponding algorithm for homogeneous data. Other than these regression models, distributed inference and federated learning algorithms have been developed for support vector machine (SVM) (66), principal component analysis (PCA) (67), latent Dirichlet allocation (LDA) (68), topic models (69), and quantile regression (70), among others.

Another framework of methods to handle between-site heterogeneity involves hierarchical models with random effects. One-shot and few-shot federated learning algorithms have been developed for fitting linear mixed effects models [DLMM algorithm (71)] and generalized linear mixed effect models [dPQL algorithm (46)], respectively. Notably, using Laplacian approximation (72), the dPQL algorithm achieves lossless results within 5 to 10 iterations, which is a significant improvement compared to an earlier algorithm built on an expectation–maximization algorithm (73, 74) that takes 1,000 iterations and could yield different results depending on different initializations (71).

For high-dimensional observational data like EHRs, federated learning algorithms for statistical and machine learning models with various penalty functions (75–79) have been developed to integrate homogeneous or heterogeneous data. Robustified versions of federated learning algorithms using simple, robust statistical summaries have been developed (80–82) to handle outlying studies within distributed research networks. Further, to aggregate causal effects across sites while accounting for between-site heterogeneity, communication-efficient federated learning algorithms have been proposed and implemented in real-world studies.

These algorithmic advances hold significant implications for biomedical research, enabling more accurate and efficient data analysis across distributed networks while accounting for between-site heterogeneity. Major research communities like OHDSI (13, 83) and the International Agency for Research on Cancer (https://www.iarc.who.int/) have adopted some of these methods, which are expected to make a lasting impact on biomedical studies and public health by facilitating more efficient and inclusive data sharing for multi-institutional collaborations.

## COMPARISONS BETWEEN CENTRALIZED AND FEDERATED DATA MODELS

Overall, centralized and federated data models share many of the same goals, but different data-sharing strategies create unique strengths for each approach (Table 1).

Both centralized and federated data models share the ability to improve result generalizability, enhance analysis power and efficiency, and promote collaborative efforts by leveraging diverse datasets from multiple institutions and health systems. Centralized data models, while demanding significant upfront investment in personnel, infrastructure, and data harmonization, distinguish themselves by providing a secure, privacy-focused environment and the widest array of available analytical methods. In contrast, federated data models strike a balance between infrastructure requirements, availability of analytical methods, privacy risks, and communication efficiency. Among various federated approaches, the iterative form aligns more closely with centralized models, requiring a moderate infrastructure setup. On the other hand, one-shot federated models stand out as the most distinct from centralized models with minimal requirements.

## SUMMARY AND FUTURE DIRECTIONS

We have reviewed the pros and cons of centralized versus federated models for the analysis of clinical data from EHRs. Each has strengths and weaknesses, and the choice of approach for any clinical research project can depend on balancing data accessibility and data security. Tremendous progress has been made over the last decade on data standards and data models for both approaches. We have also seen the implementation of numerous large-scale data projects of both types, giving us valuable information about their performance in practice. Continuous monitoring and auditing of these efforts will facilitate further improvement of each approach and additional delineation of their strengths and weaknesses. For example, how secure are centralized data? Will we see data breaches that validate a federated approach? Will federated approaches yield the insights of centralized approaches at scale?

The future of both approaches is highly dependent on factors related to data quality. Research with clinical data is currently limited by the design and construction of EHRs for billing purposes and clinical operations. Future work by health record vendors and clinicians should focus on how clinical databases can be more research friendly. For example, including the results of phenotyping algorithms for disease diagnosis in the EHR would facilitate research above and beyond ICD codes, which are often optimized for billing. This can be facilitated by the development of libraries that summarize and store algorithms for defining more accurate phenotypes (85). Further, work on standardizing structured data and unstructured clinical notes across clinicians and across EHRs would go a long way toward reducing the noise present in clinical data. This would in turn improve the power of analytical methods to identify associations and causal effects using both centralized and federated approaches. Fortunately, we have seen an increase in the number of publications addressing the quality of EHR data despite the lack of standards for doing so (86).

Central to the success of both approaches is reliance on CDMs to standardize data across different sources. Perhaps the most commonly used data model is OMOP (10), which has enabled the OHDSI consortium (13) to organize large, federated analyses of EHR data. While significant progress has been made in the area, there are important limitations that will need to be addressed in the coming years. For example, new data types from emerging technologies such as whole genome sequencing (87), wearable devices (88), and AI (89) need to be included in data models for these data to be useful. From a practical perspective, we need to find ways to make CDMs more accessible, user-friendly, and easier and cheaper to deploy. These are currently barriers for some healthcare organizations that might want to contribute data to a large collaborative project.

In addition to CDMs, biomedical ontologies can add tremendous value to data by modeling the sematic relationships between the entities captured in EHRs as well as knowledge sources. This not only improves interoperability of data across clinical sources but can also enrich data analysis through improved understanding of the data and their quality. Further, it is possible to make inferences from the data graph defined by an ontology to facilitate more accurate estimates of patient features such as weight that are inconsistently measured across multiple encounters. This functionality was realized in the PennTURBO Sematic Engine, which uses sematic web technologies to create models of clinical data (90). This can reduce noise and improve the power of data analysis. More work is needed on the development, evaluation, and application of ontologies as part of a comprehensive data modeling and management plan for both centralized and federated approaches.

The role of AI in centralized and federated data analysis will need a lot of attention in the coming years. How do we make AI tools easily available as part of centralized data resources so that all investigators can benefit from building machine learning models with complex data queries and data cleaning? How do we combine the results of machine learning analyses run across federated data sources to achieve predictive accuracies on par with those derived from centralized data? How do we most effectively use biomedical ontologies to define research questions and preprocess data for AI analysis? Further, how do we use the vast quantities of knowledge from the biomedical literature to inform AI for tasks such as feature selection, model selection, and model interpretation? The role of knowledge in AI is not new (91), but it has been underutilized in the era of deep learning.

None of this is possible without information technology support. As the size of data collections increases and AI algorithms and software become more complex, the need for high-performance computing dramatically increases. The cost of training a large language model with billions of parameters can easily exceed $1 million. Deep learning algorithms can also consume large amounts of computing resources. Recent estimates suggest that the carbon footprint of some AI analyses can be measured in tons of carbon dioxide per investigator (92). We need to work toward financially sustainable and green computing resources and technology to enable the large-scale analyses that both centralized and federated approaches allow.

Finally, it is worth mentioning the rise of synthetic data that have been enabled by AI technology. Synthetic data are simulated in a way that captures the patterns in the real clinical data but are completely artificial, thus avoiding privacy and security concerns. Future studies will need to compare and contrast the use of synthetic data in the context of both centralized and federated approaches. Do synthetic data eliminate the need for federated approaches? Will different institutions feel comfortable sharing artificial data? Can these data be made publicly available? Progress is being made in this area using generative AI algorithms (93), with results suggesting that machine learning models perform similarly between real and synthetic EHR datasets (94).

Although the debate about centralized versus federated approaches to data analysis is often presented as an either/or situation, it is very likely that both approaches will persist into the future. As such, we need investment into research that will help each approach maximize analytical power and performance to yield the new clinical insights we need for our healthcare organizations to function as learning health systems that are able to continuously improve using their data and data shared centrally or in a federated manner.

## Figures and Tables

**Table 1:** Features of centralized and federated data models

**Features**	Centralized data model	Federated data model, iterative	Federated data model, few-shots	Federated data model, one-shot
Improving result generalizability	Leveraging diverse and representative datasets enhances result generalizability.			
Improving analysis power and efficiency	Increased total sample size enhances analysis power and efficiency.			
Promoting research collaboration	Collaborative efforts among researchers benefit all data models.			
Ease of building infrastructures	Requires significant personnel, organizational trust, computational resources, and data storage infrastructures	Requires major infrastructure and major organizational trust	Requires moderate infrastructure and minimal collaborative agreement	Requires minimal infrastructure and minimal collaborative agreement
Control of the shared data	Minimal (only through the data use agreement)	Minimal (data partner can drop from a given study)	High (data partners manually review the aggregated data before sharing)	High (data partners manually review the aggregated data before sharing)
Data harmonization	Involves data ingestion and harmonization, utilizing standards like the Observational Medical Outcomes Partnership common data model to maximize analytics	Involves harmonization and standardization to common data models, yet different data partners may have different capabilities of harmonizing and standardizing their data		
Availability of analytical methods	Offers the broadest selection of analytical methods, including statistical and machine learning (ML) models and deep learning models	Operates the same set of analytic methods as in the centralized model, at the cost of high communications across data sites and potentially longer computing time	Deploys advanced statistical analysis and a wide set of ML models; limited capability of running deep learning models (research on expanding the scope of model is actively ongoing)	Deploys advanced statistical analysis and a wide set of ML models; limited capability of running deep learning models (research on expanding the scope of model is actively ongoing)
Frequency of coordination	Involves data ingestion once or at regularly scheduled intervals (e.g., quarterly updates of data)	Involves frequent coordination for multiple rounds of communication and model refinement	Requires a few instances of coordination for enhanced communication; may suffer from synchronization problems, especially with a moderate or large number of data partners	Only requires a one-time coordination; does not suffer from synchronization problems
Data privacy	Data are stored and protected by the coordinating center	Privacy exposure risk scales with the number of iterations of sharing aggregated data	Presents lower risks of data privacy exposure, as aggregated data are transferred fewer times	Minimal risk of data privacy exposure, as information is communicated only once

## Abbreviations

Electronic health record (EHR): a digital version of a patient’s clinical record that contains their medical history, diagnoses, medications, laboratory test results, and other clinical information
Precision medicine: tailoring medical treatment and interventions to the genes, environments, and lifestyles of each patient
Centralized database: data collected from multiple institutions are managed and stored in a single, central location
Federated database: data are stored distributedly across multiple institutions; results or data summaries may be shared without transferring individual-level data across institutions
Common data model (CDM): a standardized structure for organizing and representing data
Biobank: a repository that stores and manages biological samples collected from individuals for use in biomedical research
Patient heterogeneity: the variability among individuals in a patient population, which is influenced by factors such as demographics, clinical characteristics, treatment response, etc.
Iterative federated learning model: a federated learning approach involving multiple rounds of information transfer between sites
Individual-level data: datasets that contain information on each individual participant in a study
Pharmacoepidemiologic: related to the study of the use and effects of drugs in large populations in real-world conditions
Pharmacovigilance: the practice of collection, detection, assessment, monitoring, and prevention of adverse drug effects
Ontology: a representation of knowledge that includes definitions and relationships among concepts within a domain
Synthetic data: artificially generated data that mimic real data but do not contain information about specific individuals
